# A comparison of average wages with age-specific wages for assessing indirect productivity losses: analytic simplicity versus analytic precision

**DOI:** 10.1007/s10198-016-0819-9

**Published:** 2016-07-14

**Authors:** Mark P. Connolly, Cole Tashjian, Nikolaos Kotsopoulos, Aomesh Bhatt, Maarten J. Postma

**Affiliations:** 1 Unit of PharmacoEpidemiology and PharmacoEconomics, Department of Pharmacy, University of Groningen, Antonius Deusinglaan 1, 9713 AV Groningen, The Netherlands; 2 Global Market Access Solutions (GMAS), St-Prex, Switzerland; 3 John Radcliffe Hospital, Oxford, OX3 9DU UK; 4 Institute of Science in Healthy Aging and Healthcare (SHARE), University Medical Center Groningen (UMCG), Groningen, The Netherlands; 5 Department of Epidemiology, University Medical Center Groningen (UMCG), Groningen, The Netherlands

**Keywords:** Indirect costs, Productivity, Age-specific wages, Average wages, B40

## Abstract

**Objectives:**

Numerous approaches are used to estimate indirect productivity losses using various wage estimates applied to poor health in working aged adults. Considering the different wage estimation approaches observed in the published literature, we sought to assess variation in productivity loss estimates when using average wages compared with age-specific wages.

**Methods:**

Published estimates for average and age-specific wages for combined male/female wages were obtained from the UK Office of National Statistics. A polynomial interpolation was used to convert 5-year age-banded wage data into annual age-specific wages estimates. To compare indirect cost estimates, average wages and age-specific wages were used to project productivity losses at various stages of life based on the human capital approach. Discount rates of 0, 3, and 6 % were applied to projected age-specific and average wage losses.

**Results:**

Using average wages was found to overestimate lifetime wages in conditions afflicting those aged 1–27 and 57–67, while underestimating lifetime wages in those aged 27–57. The difference was most significant for children where average wage overestimated wages by 15 % and for 40-year-olds where it underestimated wages by 14 %.

**Conclusions:**

Large differences in projecting productivity losses exist when using the average wage applied over a lifetime. Specifically, use of average wages overestimates productivity losses between 8 and 15 % for childhood illnesses. Furthermore, during prime working years, use of average wages will underestimate productivity losses by 14 %. We suggest that to achieve more precise estimates of productivity losses, age-specific wages should become the standard analytic approach.

## Background

Assessing the broader consequences of disease is often considered an important element of economic appraisals in health. Health economic appraisals frequently take a wider societal perspective, thus the quantification of productive output or productivity losses (i.e., indirect costs resulting from a disease) is fundamental to understanding the impact of health outcomes. There are several methods which have been used historically to quantify the loss of productive output, including the human capital approach which has been used very frequently in capturing indirect costs associated with a disease. The human capital approach uses gross wages as a proxy for productivity, although simply applying labor wage rates is likely to underestimate societal losses, as workers actually generate more value than purely reflected in their actual wages, as firms take profits from productive output. In applying wages to estimate productivity losses and a function of decreased productivity (e.g., absenteeism, presenteeism), limited guidance is available regarding the choice of wages to include in the analysis. This explains the variation in how indirect costs are often calculated, which ranges from using average wages [1–5], GDP per capita [6–10], age-specific wages [11–13], and in some cases not specifying how labor was valued [14–16]. Choosing the most appropriate wage data is especially important when considering the impact that productivity costs can have on cost-effectiveness thresholds.

In this methodological concept note, we compare two of the more common implementation methods of the human capital approach for calculating productive output loss; namely, the use of average wages versus the use of age-specific wages applied to morbidity and mortality events. While average wages are often easier to identify in the public domain, they do not reflect the established life cycle of wages which is better reflected using age-specific wage rates. The wage life cycle was established in human capital economic theory since workers usually start their career with low wages, see an increase as they gain more experience and expertise, and finally usually see a decrease in wages as they often begin working less in old age, and their employers are less incentivized to invest in older employees [17]. Due to the consistency and significance of the variance of earnings over a lifetime, age-specific wages are able to capture lifetime earnings projections more accurately. We observe in the published literature that investigators often arbitrarily use either average wage or age-specific wages to estimate indirect costs without justification for such choices. Our analysis attempts to demonstrate how the use of each wage rate might influence the estimation of lifetime productive output based on human capital economics, and hence can be used as a tool to aid analysts to better understand the degree to which age-specific wage data is superior and how wages might need to be adjusted to reflect the true lifetime productive output which illnesses may influence.

## Methods

To compare the difference in productivity losses we identified age-specific wage (2013–2014) data in 5-year increments over an individual’s working life, and average wages for the United Kingdom [18]. Lifetime wage estimations for an average individual were produced for a newborn over his/her lifetime and subsequently the same estimation was produced for different starting ages (e.g., a 45- or a 60-year-old permanently leaving the workforce). In the present study, we sought to compare how use of average wage rates with age-specific wage rates could influence productivity loss estimates attributed to reduced work force participation due to morbidity or mortality. For the nature of this exercise, the specific illness to which productivity losses would apply was not important, rather the incremental indirect cost estimates that can occur at different stages of life based on use of different wage rates attributed to any health condition that can lead to productivity losses.

A polynomial interpolation was used to convert 5-year wages data into annual age-specific wages estimates. A discount rate of 3 % was applied to the projected age-specific and average wage losses. A scenario analysis using discount rates of 0 and 6 % were examined to estimate the impact on the gap between age-specific and average wages. Four metrics of productive output were generated for comparison: undiscounted age-specific wages, discounted age-specific wages, undiscounted average wages, and discounted average wages. The average retirement age used was 67 years for projected wage losses. In order to compare the difference in accuracy between average and age-specific wage data at different stages of life, the percentage by which average wage varies from average lifetime wages by age group was calculated. This provided insight into whether average wages data overestimated or underestimated lifetime productive output for different age groups.

## Results

**Fig. 1:**
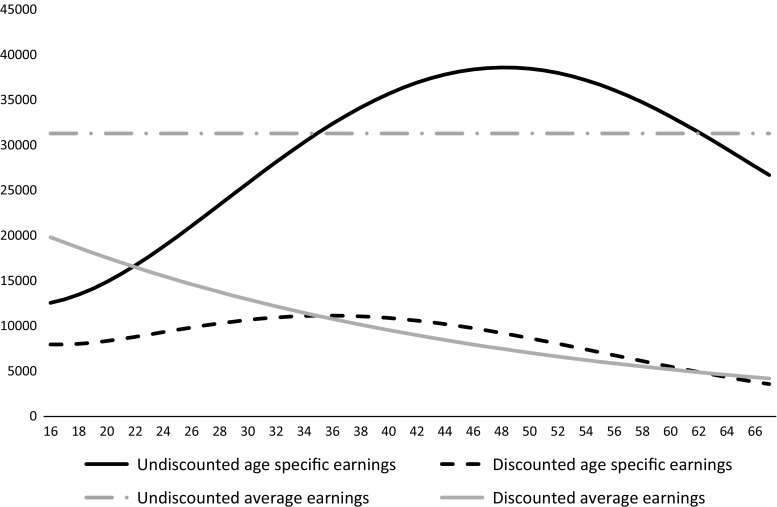
Illustration of discounted and undiscounted productivity loss estimates obtained in a 1-year-old child using average wages and age-specific wages

**Fig. 2:**
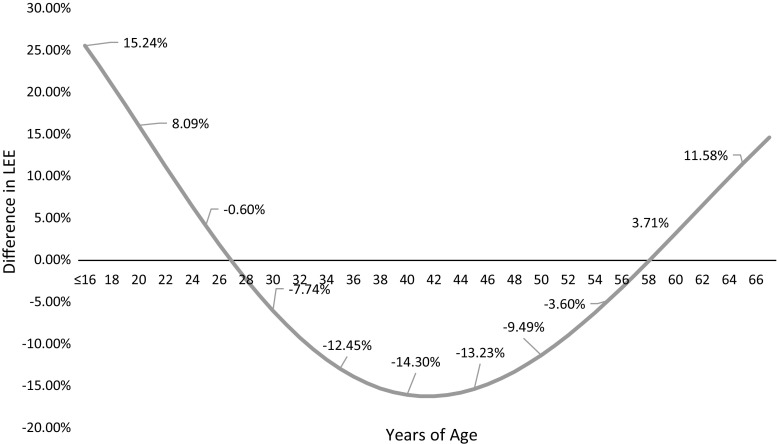
Difference in lifetime earnings estimation (LEE) age-specific/average wage

**Fig. 3:**
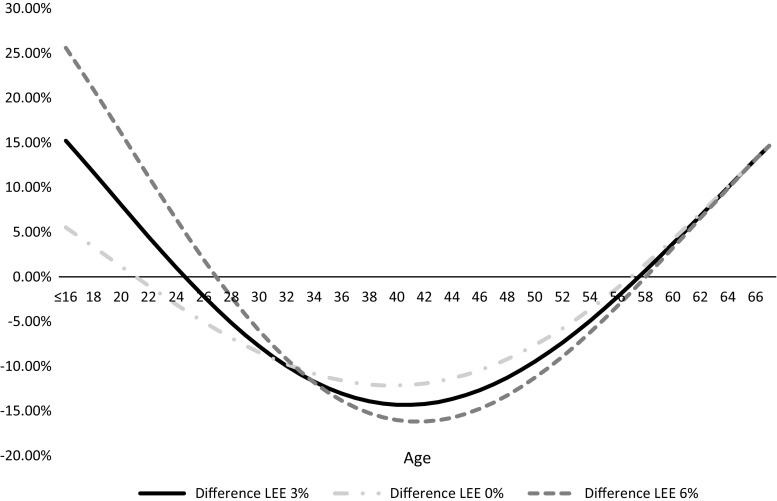
Difference in lifetime earnings estimation (LEE) age-specific/average wage by discount rate

Figure 1 illustrates the average wage and age-specific indirect cost calculations for an illness that may affect a newborn child in the UK over their projected lifetime. In the base analysis costs were discounted at 3 %. Figure 2 depicts the corresponding percentage difference in the estimated lifetime productive output for different starting ages of a health condition. From age 1–25 and 57–67, using average wage will likely overestimate lifetime productive output. Likewise, using average wages for those between 25 and 56 years of age will underestimate lifetime productive output. The difference in lifetime wages estimation can be thought of as a margin of error incurred by using average wage data to estimate lost potential earnings: if one was to estimate lifetime wage losses of a 25- or 57-year-old, the margin of error would be near zero. Using average wage to estimate lifetime earning potential of a child or a 40-year-old, however, could lead to an overestimation of 15.24 % or an underestimation of 14.3 %, respectively. Figure 3 depicts the incremental between curves calculated using different discount rates. The higher the discount rate used, the higher the margin of error for estimating the future potential earnings of a young adult or child. For example, for a childhood health condition with permanent disability at 0 % discount rate the average wage overestimates indirect costs by 5.5 %. When 3 and 6 % discount rates are applied using average wages, indirect costs are overestimated by 13.5 and 23.3 %, respectively. The data in Fig. 3 is for illustrative purposes and will vary depending on the age of illness onset and discount rate as depicted in the graph. However, over time, the discount rate used has less of an impact on the incremental difference between average wage and age-specific wages, with the curves converging in older workers, i.e., those over 58 years of age.

## Discussion

The analysis described here illustrates the disadvantages associated with using average wages to reflect lifetime productivity losses, and thus indirect costs, using the human capital approach. Whilst average wage data offers analytic simplicity, and is easier to identify for many countries, it does not adequately reflect indirect productivity losses attributed to morbidity and mortality. In fact, our analysis indicates that the use of average wages either overestimates or underestimates lost productive output over a lifetime depending on the age at which the health impact occurs. Age-specific wages reflect many key elements of the labor market over a lifetime, including the accumulation of knowledge, changes in productivity, and specialization of skills, allowing variation in ability to barter wages. Failing to consider these shifts in wages over a life cycle can lead to significant inaccuracies in estimating indirect costs of morbidity and mortality.

One could argue that the differences described here may be inconsequential in many analyses, particularly considering that only a limited number of countries take into consideration productivity loss or indirect costs in reimbursement decisions that influence formulary access. However, previous studies [2] have shown that the inclusion of indirect costs can influence the likelihood that a product may be cost-effective or not. In some cases, this simple methodological adjustment could even change the viability of policy choices by altering their projected impact. Bearing in mind that the inclusion of indirect costs may change conclusions on cost-effectiveness underlines the importance of adequately calculating these costs. The findings described here would also be relevant for investigators applying the friction cost approach. The message described here is focused on the choice of wages, and not about the time estimates attributed to lost work from disease.

Since the introduction of labor wage rates to decision making, analysts have sought to understand the broader consequences of health. Few would dispute that labor wage rates are a poor proxy for value because they do not account for other forms of economic contribution an individual can bring to society; however, they can be implemented to grasp a more complete understanding of the indirect costs of illness, thereby increasing the accuracy of the value of treatment. To ensure the highest possible degree of accuracy when estimating indirect costs using these methods, the metric used to measure wage is key. Age-specific wages should be used in most circumstances whenever available; if not, the resulting imprecision of using average wages data must be taken into account. In the absence of age-specific data, the relationship between age-specific wages and average wages described here may offer a factor adjustment that allows for improved precision.

The life cycle of wages reflected by age-specific wages is well established in published literature [19]. The consistency of this relationship suggests that the results described here are applicable to other countries, highlighting the importance of using age-specific wages more broadly. Although the exact degree to which average wage data misestimates lifetime earnings will vary across countries, the general curve of earnings over a lifetime is consistent across similar countries. This knowledge should be considered when choosing a metric to project potential income or calculate the present value of lost lifetime earnings as an indirect societal cost of death by illness.

## References

[1] Izquierdo G, Torres JP, Santolaya ME, Valenzuela MT, Vega J, Chomali M (2015). Cost-effectiveness analysis of a multicomponent meningococcal serogroup B vaccine in hypothetic epidemic situation in a middle-income country. Hum. Vaccin. Immunother..

[2] Oliva J, Lobo F, López-Bastida J, Zozaya N, Romay R (2005). Indirect costs of cervical and breast cancers in Spain. Eur J Health Econ..

[3] Gregg M, Meier G (2014). Protecting productivity with quadrivalent inactivated influenza vaccine in the UK. Value Health.

[4] Sander B, Gyldmark M, Hayden FG, Morris J, Mueller E, Bergemann R (2005). Influenza treatment with neuraminidase inhibitors. Eur J Health Econ..

[5] Akweongo P, Dalaba MA, Hayden MH (2013). The economic burden of meningitis to households in Kassena-Nankana District of Northern Ghana. PLoS One.

[6] Hanly P, Soerjomataram I, Sharp L (2014). Measuring the societal burden of cancer: the cost of lost productivity due to premature cancer-related mortality in Europe. Int J Cancer.

[7] Wu DB-C, Chang C-J, Huang Y-C, Wen Y-W, Wu C-L, Fann CS-J (2012). Cost-effectiveness analysis of pneumococcal conjugate vaccine in Taiwan: a transmission dynamic modeling approach. Value Health.

[8] Garattini L, Tediosi F, Ghislandi S, Orzella L, Rossi C (2000). How do Italian pharmacoeconomists evaluate indirect costs?. Value Health..

[9] Soarez PCD, Sartori AMC, Nóbrega LDAL, Itria A, Novaes HMD (2011). Cost-effectiveness analysis of a universal infant immunization program with meningococcal C conjugate vaccine in Brazil. Value Health..

[10] Ozawa S, Clark S, Portnoy A, Grewal S, Brenzel L, Walker DG (2016). Return on investment from childhood immunization in low- and middle-income countries 2011–2020. Health Aff..

[11] Kotsopoulos N, Connolly MP, Remy V (2015). Quantifying the broader economic consequences of quadrivalent human papillomavirus (HPV) vaccination in Germany applying a government perspective framework. Health Econ Rev.

[12] Yang B-M, Kim DJ, Byun KS, Kim HS, Park J-W, Shin S (2009). The societal burden of HBV-related disease: South Korea. Dig Dis Sci..

[13] Hemels M, Kasper S, Walter E, Einarson T (2004). Cost-effectiveness analysis of escitalopram: a new SSRI in the first-line treatment of major depressive disorder in Austria. Curr. Med. Res. Opin..

[14] Sobocki P, Ekman M, Agren H, Jönsson B, Rehnberg C (2006). Model to assess the cost-effectiveness of new treatments for depression. Int. J. Technol. Assess. Health Care.

[15] Valenstein M, Vijan S, Zeber J, Boehm K, Buttar A (2001). The cost-utility of screening for depression in primary care. Ann. Intern. Med..

[16] Demyttenaere K, Hemels M, Hudry J, Annemans L (2005). A cost-effectiveness model of escitalopram, citalopram, and venlafaxine as first-line treatment for major depressive disorder in Belgium. Clin. Ther..

[17] Krol M, Papenburg J, Koopmanschap M, Brouwer W (2011). Do productivity costs matter?. PharmacoEconomics..

[18] Distribution of median and mean income and tax by age range and gender, 2013–2014. GOV.UK 2016. https://www.gov.uk/government/statistics/distribution-of-median-and-mean-income-and-tax-by-age-range-and-gender-2010-to-2011. Accessed Mar 15, 2016

[19] OECD Employment Outlook 1998. OECD Employment Outlook. 1998:123–151. http://www.oecd.org/els/emp/2080254.pdf. Accessed Mar 30, 2016

